# An Audit on Medical Seclusion Reviews in a Psychiatric Intensive Care Unit

**DOI:** 10.1192/bjo.2026.11655

**Published:** 2026-06-30

**Authors:** Punya Mulky, Sasha Kojo Daniels, Sulagna Saha, Raghu Vutla

**Affiliations:** 1 Bradford District Care NHS Foundation Trust, Bradford, United Kingdom; 2 Leeds and York Partnership NHS Foundation trust, Leeds, United Kingdom; 3 North East London NHS Foundation trust, London, United Kingdom; 4 South West Yorkshire Partnership NHS Foundation Trust, Wakefield, United Kingdom

## Abstract

**Aims:**

The South West Yorkshire Partnership NHS Foundation trust (SWYT) policy on seclusion and long term segregation refers to Seclusion as ‘supervised confinement and isolation of a service user, away from other service users, in an area from which the service user is prevented from leaving, where it is of immediate necessity for the purpose of the containment of severe behavioural disturbance which is likely to cause harm to others.’ It is essential that they are afforded the procedural safeguards of the Mental Health Act Code of Practice. The SWYT trust policy provides clear recommendations on medical seclusion reviews.

The aim of the audit is to check the adherence to SWYT trust policy on medical seclusion reviews in a Psychiatric Intensive care Unit (PICU).

**Methods:**

The SWYT trust policy on medical seclusion reviews has a seclusion toolkit which outlines the following timeframes for assessment: Initial medical review within an hour of seclusion, 4-hourly medical reviews till first Multidisciplinary (MDT) meeting, first MDT, independent MDT within 8-18 hours of seclusion, regular reviews thereafter (included 1 medical (non-RC) review, 1 Responsible Clinician (RC) review and 1 MDT till the end of seclusion. The RC MDT and RC medical review may be combined). The policy needs to be followed for seclusions initiated during out of hours/ weekends/bank holidays.

The list of all new seclusions in PICU from January 2025 to April 2025 was obtained from the Trust Performance and Business Intelligence team. An audit tool was prepared using the seclusion toolkit in the trust policy. The audit tool comprised of questions checking if the medical, MDT and Independent reviews were completed in a timely manner as per the trust guidance.

Data collection was done by members of the audit team. All the data was collected from electronic records.

**Results:**

***Key successes:***

1. In 92.7% cases, first MDT was completed within 08:00 to 22:00 the next day.

2. 95% seclusion reviews on bank holidays/ weekend and out of hours were adherent to the trust policy.

3. Non-RC medical reviews were completed daily for the duration of the seclusion in 87.2% cases.

***Key concerns / areas for improvement:***

1. Only 24.2% completion of independent MDT reviews during a period of seclusion.

2. 64.4% resident doctor reviews are done within 1 hour of commencement of seclusion.

3. Daily MDT reviews were completed in only 66.6% cases. This was noted to be due to incorrect documentation on the seclusion proforma.

**Conclusion:**

This audit highlights high compliance for first MDTs and out of hours reviews but also highlights areas for improvement such as low rates of completion of independent MDT and initial 1 hour reviews. Recommendations included prompts for independent MDT reviews, including seclusion toolkit in new doctors’ induction training and discussing with Responsible Clinicians about accurate documentation of daily seclusion reviews.

Seclusion is a restrictive procedure and therefore ensuring adherence to trust guidelines on seclusion reviews is important.

